# Insights from the Construction of Adenovirus-Based Vaccine Candidates against SARS-CoV-2: Expecting the Unexpected

**DOI:** 10.3390/v15112155

**Published:** 2023-10-25

**Authors:** Denice Weklak, Julian Tisborn, Maurin Helen Mangold, Raphael Scheu, Harald Wodrich, Claudia Hagedorn, Franziska Jönsson, Florian Kreppel

**Affiliations:** 1 Chair of Biochemistry and Molecular Medicine, Center for Biomedical Education and Research (ZBAF), Witten/Herdecke University, Stockumer Str. 10, 58453 Witten, Germany; denice.weklak@uni-wh.de (D.W.); julian.tisborn@uni-wh.de (J.T.); maurin.mangold@t-online.de (M.H.M.); raphael.zhili@gmail.com (R.S.); claudia.hagedorn@uni-wh.de (C.H.); 2 Microbiologie Fondamentale et Pathogénicité, MFP CNRS UMR 5234, Université de Bordeaux, 33076 Bordeaux, France; harald.wodrich@u-bordeaux.fr

**Keywords:** adenovirus, viral vector, vaccine, SARS-CoV-2, spike protein

## Abstract

To contain the spread of the SARS-CoV-2 pandemic, rapid development of vaccines was required in 2020. Rational design, international efforts, and a lot of hard work yielded the market approval of novel SARS-CoV-2 vaccines based on diverse platforms such as mRNA or adenovirus vectors. The great success of these technologies, in fact, contributed significantly to control the pandemic. Consequently, most scientific literature available in the public domain discloses the results of clinical trials and reveals data of efficaciousness. However, a description of processes and rationales that led to specific vaccine design is only partially available, in particular for adenovirus vectors, even though it could prove helpful for future developments. Here, we disclose our insights from the endeavors to design compatible functional adenoviral vector platform expression cassettes for the SARS-CoV-2 spike protein. We observed that contextualizing genes from an ssRNA virus into a DNA virus provides significant challenges. Besides affecting physical titers, expression cassette design of adenoviral vaccine candidates can affect viral propagation and spike protein expression. Splicing of mRNAs was affected, and fusogenicity of the spike protein in ACE2-overexpressing cells was enhanced when the ER retention signal was deleted.

## 1. Introduction

Since emerging in Wuhan, China, in late 2019, severe acute respiratory syndrome coronavirus 2 (SARS-CoV-2) has caused over 6.9 million confirmed deaths worldwide (https://covid19.who.int/, accessed 28 August 2023) to August 2023 and severely affected the world economy [1]. Once the World Health Organization (WHO) declared SARS-CoV-2 a pandemic in March 2020, researchers strove for the quick development of novel vaccines and drugs to combat the spread of coronavirus disease 2019 (COVID-19). Because of these urgent circumstances, vaccination platforms that allow rapid engineering and simultaneously deliver a robust immune response, such as the mRNA or adenoviral vaccination platforms, asserted themselves against classical vaccination regimes such as live-attenuated vaccines. Adenoviral and mRNA vaccination platforms allow immediate reaction to new pathogens or strains by altering the sequence of the to-be-delivered nucleic acid. This diversity in vaccine regimens was one major tool controlling the spreading of the pandemic. In particular, adenoviral vaccines offer several advantages as an emergency outbreak vaccination platform, such as high target cell transduction and gene transfer efficacy, as well as triggering robust humoral and cellular immune responses and prolonged stability at room temperature for storage [2]. The design of such adenoviral vectors for the expression of a specific transgene is not trivial, as the expression of particular transgenes may affect viral propagation and may thus require a protein-dependent design of the expression cassette. For efficient vaccine production, optimal vector design relies on the perfect balance between transgene expression and vector functionality.

Here, we want to share our knowledge gained by generating a series of human adenovirus type 5 (HAdV-C5) vector-based vaccine candidates. While a majority of adenoviral vaccines that gained conditional market approval are based on tetracycline-controlled gene expression systems [3,4], our vector design relies on constitutive protein expression in transduced target cells. This way, effects of transgene expression, such as antigen-related toxicity on human cells, can be observed, enabling early estimation of patient safety following vaccination. SARS-CoV-2 is an ssRNA virus, and the spike protein is encoded by RNA. Therefore, SARS-CoV-2 RNA is never confronted with the RNA-processing machinery in the nucleus but is immediately translated in the cytoplasm. To clone the spike gene into an adenoviral genome, a DNA sequence originating from the RNA sequence has to be generated. Consequently, this DNA (of a sequence never meant for being localized in a nucleus) needs to be transcribed and processed before leaving the nucleus. As such, transferring the sequence of a genuine RNA gene to a DNA vector bears the risk of aberrant splicing and, as a consequence, defective transgene products. Therefore, obtaining a substantial understanding regarding the contextualizing of genes from RNA viruses into a DNA background is an essential basis for future developments of adenoviral vaccines.

With this study, we want to share our knowledge gained while generating our own set of adenovirus type 5 vector-based vaccine candidates harboring a CMV (cytomegalovirus)promoter-driven *spike* expression cassette located within the HAdV-C5 E1-region. We report that physical titers, viral propagation and spike protein expression were affected based on different expression cassette designs. While mRNA splicing of transcripts was seemingly not affected, fusogenicity of ACE2 (angiotensin-converting enzyme)-overexpressing cells was enhanced, dependent on the design of adenoviral vectors. We think that these insights into our struggles with vector production will be useful for future vaccine development.

## 2. Materials and Methods

### 2.1. Cell Lines and Cell Culture

HEK293 (ATCC^®^, Manassas, VA, USA, CRL-1573™) and A549 (ATCC^®^, Manassas, VA, USA, CLL-185™) cells were cultured in MEM Eagle (PAN Biotech™, Aidenbach, Germany, P04-08500) supplemented with 10% FCS (PAN Biotech™, Aidenbach, Germany, P40-37500) and 1% penicillin–streptomycin (PAN Biotech™, Aidenbach, Germany, P06-07050). U2OS-GFP-ACE2 cells were generated following transduction and selection with a lentivirus encoding the SARS-CoV-2 receptor angiotensin-converting enzyme 2 (ACE2, addgene, Watertown, MA, USA, #145839) and maintained under selection of 5 µg/mL puromycin (Merck KGaA, Darmstadt, Germany, P7255-25MG). U2OS, as well as KM-12 (Cellosaurus, CVCL_1331), Huh-7 (Cellosaurus, CVCL_0336) and SH-SY5Y (ATCC^®^, Manassas, VA, USA, CRL-2266™) were maintained in DMEM (PAN Biotech™, Aidenbach, Germany, P04-03590) supplemented with 10% FCS and 1% penicillin–streptomycin (PAN Biotech™, Aidenbach, Germany, P06-07050). Cells were passaged twice per week with 0.05% trypsin–0.02% EDTA (PAN Biotech™, Aidenbach, Germany, P10-0231SP).

### 2.2. Vector Construction

For this study, each E1/E3-deleted vector of human adenovirus type 5 (AY339865; bp 1–440, bp 3522–28131, bp 30814–35934) was generated using the Counter-Selection BAC Modification Kit (GeneBridges GmbH, Heidelberg, Germany, K002), which is based on pRed/ET recombination. Briefly, using in vivo homologous recombination in *Escherichia coli* (*E. coli*) DH10β, a counter-selection rpsL-neo cassette with flanking homology arms was introduced in an E1-located expression cassette consisting of a CMV promoter and an SV40 intron. The rpsL-neo cassette was either replaced by the Wuhan isolate (GenScript Biotech Corporation, Piscataway Township, NJ, USA, NC_045512.2) or codon-optimized (pUC57-2019-nCOV-S, GenScript Biotech Corporation, Piscataway Township, NJ, USA) DNA sequence of the full-length spike protein or only the S_1_ domain. Furthermore, the SV40 intron, the N-terminally located signal peptide (SP) of the spike protein, or the endoplasmic reticulum (ER) retention signal of the spike protein was deleted by placement of the rpsL-neo cassette and subsequently replaced by a non-coding non-intronic sequence in the case of the intron or by the IL-2 signal peptide in the case of the spike signal peptide (for reference, see Section 3.1).

### 2.3. Adenovirus Vector Purification

Before transfection of cells, adenoviral vectors were liberated from bacterial bacmid backbone by restriction digestion with SwaI (New England Biolabs GmbH, Ipswich, MA, USA, R0604S). In short, 2.5 µg of DNA was incubated with 3 U SwaI in supplied buffer at 25 °C overnight. HEK293 cells were seeded at a density of 1 × 10^5^ cells/well in a 24-well plate and cultivated overnight. The next day, 500 ng DNA was premixed with a 150 mM NaCl solution to a volume of 25 µL, and 6 µL of a 7.5 mM linear polyethyleneimine (PEI) (22 kDa) solution was mixed with a 150 mM NaCl solution to a volume of 25 µL. PEI mixture was added to the DNA solution and incubated at room temperature (RT) for 10 min. Subsequently, the complete PEI-DNA mixture was added to one well of the 24-well plate. Cells were harvested once cytopathic effects became visible after roughly 12 to 14 days. Lysates were used for cycles of reinfections and vector particles were finally purified using CsCl density gradient centrifugation. Two purification steps using discontinuous CsCl gradients (1.27 g/cm^3^ and 1.41 g/cm^3^) were performed, and vector bands were collected after centrifugation at 4 °C and 13.200 rpm for 2 h. Preparations were desalted using PD10 columns (Cytiva, Marlborough, MA, USA, 17085101). For the determination of physical titers, 20 µL of vector preparation was mixed with 0.1% SDS and 79 µL buffer and incubated at 56 °C for 10 min. Extinction at 260 nm was measured using UV spectroscopy, and titers were calculated utilizing the following formula:

vp/µL = E_260_ × 1.1 × 10^9^ × DF (1)

where vp = virus particles, E_260_ = extinction at 260 nm, and DF = dilution factor

### 2.4. Polymerase Chain Reaction (PCR)

DNA fragments for homologous recombination were generated using Q5^®^ high-fidelity polymerase (New England Biolabs GmbH, Ipswich, MA, USA, M0491L) with primers including 50 bp homology arms to the insertion site. Thus, 10 ng DNA template were mixed with 0.04 U/µL polymerase, 0.2 mM dNTPs, 1 µM primers forward and reverse (Table S1), as well as the appropriate buffer. The following program was used: cycle 1—98 °C, 120 s; cycles 2–27—98 °C, 30 s, then 67 °C, 30 s, followed by 72 °C, 120 s; cycle 28: 72 °C, 180 s. Colony PCR was performed to verify the replacement of rpsL-neo cassette by target sequence. As such, picked colonies were cultured in 100 µL LB medium supplemented with respective antibiotics and incubated at 37 °C for 3 h. Thereafter, 3 µL of bacterial culture was added to a mixture of 1 µM forward and reverse primers (forward: 5′ GCTCGTTTAGTGAACCGTCAGA 3′, reverse: 5′ GAGGCCGAGTTTGTCAGAAAGC 3′), 0.2 mM dNTPs, 0.05 U/µL GoTaq^®^ polymerase (Promega Corporation, Madison, WI, USA, M3001) in appropriate buffer. PCR reaction was performed with the following program: cycle 1—95 °C, 180 s; cycles 2–31—95 °C, 45 s, then 58 °C, 45 s, followed by 74 °C, 120 s; cycle 32—74 °C, 500 s. For the analysis of mRNA splicing of transcripts, a 3 µL DNA sample was mixed with 0.2 mM dNTPs, 0.05 U/µL GoTaq^®^ polymerase (Promega Corporation, Madison, WI, USA, M3001), and 1 µM forward and reverse primers (Table S2) in appropriate buffer. PCR cycles were: cycle 1—95 °C, 180 s; cycles 2–31—95 °C, 70 s, then 59 °C, 70 s, followed by 74 °C, 300 s; cycle 32—74 °C, 600 s.

### 2.5. Sodium Dodecyl Polyacrylamide Gel Electrophoresis (SDS-PAGE)

For adjustment of titer from viral preparations, 5 × 10^9^ viral particles (vp), as calculated by physical titers, were diluted in Ad buffer (150 mM NaCl, 50 mM HEPES, pH 8.0) to a final volume of 20 µL. SDS loading buffer (5 µL) was added, and samples were boiled for 5 min at 95 °C. Samples were loaded onto an 8% polyacrylamide gel, which after electrophoresis was stained using silver staining (see Section 2.6).

Spike protein expression by viral vectors was analyzed from either supernatant or cell pellet. Thus, 100 µL of supernatant after infection of HEK293 or transduction of A549 or U2OS cells was transferred to a reaction tube. Protein extraction from cells was performed according to a modified protocol of the original by K. K. Wang [5]. Cells were washed once with 1 mL DPBS (PAN Biotech™, Aidenbach, Germany, P04-36500), then treated with 300 µL RIPA-SDS buffer with protease-inhibitor cocktail (Merck KGaA, Darmstadt, Germany, 11836153001) and incubated at room temperature for 10 min. After addition of 100 µL 100% trichloroacetic acid (TCA), the supernatant was transferred to a reaction tube. The pellet after centrifugation (RT, 5000× *g*, 5 min) was washed with 1 mL 2.5% TCA and again centrifuged (RT, 5000× *g*, 5 min). Tris base (20 µL, 3 M) was added to the pellet after centrifugation, and samples were then incubated at room temperature for 30 min. Finally, samples were mixed with 20 µL ddH_2_O and 10 µL of 5× SDS sample buffer, then incubated at 95 °C for 10 min. Samples were loaded onto an 8% polyacrylamide gel, and separated proteins, after electrophoresis, were thereafter transferred to a nitrocellulose membrane (see Section 2.9).

### 2.6. Silver Staining of Polyacrylamide Gel

Silver staining of polyacrylamide gels was performed according to H. Blum et al. [6].

Briefly, polyacrylamide gel after electrophoresis was transferred into a fixation solution (50% (*v*/*v*) methanol, 12% (*v*/*v*) acetic acid, 13.6 mM formaldehyde) and incubated at room temperature for 30 min. The gel was then rinsed with a washing buffer (50% ethanol) for 15 min and then treated with pretreatment buffer (0.8 mM sodium thiosulfate) for 1 min. After washing thrice for 20 s with ddH_2_O, the gel was incubated in impregnation buffer (0.2% (*w*/*v*) silver nitrate, 13.6 mM formaldehyde) for 20 min at room temperature. Gel was subsequently developed in developer solution (6% (*w*/*v*) sodium carbonate, 16 µM sodium thiosulfate, 13.6 mM formaldehyde) after washing twice with ddH_2_O for 20 s. The reaction was stopped by treatment with stopping buffer (50% (*v*/*v*) methanol, 12% (*v*/*v*) acetic acid) after washing with ddH_2_O twice for 2 min.

### 2.7. Infection of HEK293 Cells with Adenoviral Vectors

For real-time quantitative PCR (RT-qPCR) analysis, 3 × 10^4^ HEK293 cells/well were seeded in a 96-well plate and cultured overnight. The next day, cells were infected with spike protein expressing HAdV-C5 vectors with a multiplicity of infection (MOI) of 200 according to adjusted titers and incubated for 48 h at 37 °C, 5% CO_2_. For the analysis of mRNA splicing, 2 × 10^6^ HEK293 were seeded in 6 cm cell culture dishes and cultured overnight. The following day, cells were infected with an MOI of 300 and incubated at 37 °C and 5% CO_2_ for 48 h.

### 2.8. Transduction of A549 or U2OS Cells with Adenoviral Vectors

For the analysis of mRNA splicing, 2 × 10^6^ A549 cells were seeded in 6 cm cell culture dishes a day prior to transduction. Cells were then transduced with an MOI of 300 and incubated at 37 °C and 5% CO_2_ for 48 h. 

For Western blot analysis of spike protein expression, 2 × 10^5^ U2OS cells/well were seeded in a 24-well plate and cultured overnight. Cells were transduced with spike protein encoding viral vectors using an MOI of 300 according to adjusted titers and incubated for 48 h at 37 °C, 5% CO_2_. Furthermore, to enhance understanding of spike protein expression, 2 × 10^5^ A549 cells/well in a 24-well plate were transduced with adenoviral vectors with an MOI of 1000 according to adjusted titers and incubated for 48 h at 37 °C, 5% CO_2_.

### 2.9. Fast Protein Liquid Chromatography (FPLC) for Spike Protein Purification

In order to purify glycosylated S_1_ protein from media after secretion, at least 10 × 15 cm cell culture dishes of A549 cells were transduced with appropriate vector using an MOI of 3000. The next day, cells were washed thrice with 15 mL of warm medium and DPBS. Cells were then treated with 25 mL EX-CELL^®^ (Merck KGaA, Darmstadt, Germany, 14571C-1000ML) medium and incubated at 37 °C, 5% CO_2_ for an additional 48 h until the medium was harvested. The supernatant was mixed with cOmplete™ protease-inhibitor cocktail (Merck KGaA, Darmstadt, Germany, 11697498001), then centrifuged for 8 min at 300× *g*. The supernatant was transferred into centrifuge tubes, and 5 µL [0.015 U/µL] of an avidin solution (IBA Lifesciences GmbH, Göttingen, Germany, 2-0204-050) was added. After incubation at room temperature for 20 min, tubes were centrifuged at 18.000× *g* and 4 °C for 30 min.

For protein purification, an NGC Quest 10 chromatography system (Bio-Rad Laboratories GmbH, Hercules, CA, USA, #7880001) was used. Subsequently, the supernatant was loaded onto a StrepTactin™ column (Cytiva, Marlborough, MA, USA, 29048653). Purification of S_1_ protein from the column was performed according to the manufacturer’s instructions. Product fractions after purification were concentrated to a total volume of 100 µL using Amicon^®^ ultra centrifugal units (Merck KGaA, Darmstadt, Germany, UFC503096) according to the product’s manual.

### 2.10. Western Blot

After subjecting protein samples to SDS-PAGE, proteins were transferred to a nitrocellulose membrane (Cytiva, Marlborough, MA, USA, 10600003). Membranes were blocked with 5% bovine serum albumin in TBS-T (Tris-buffered saline, 100 mM Tris and 150 mM NaCl, with 0.05% Tween-20) overnight at 4 °C. For this study, either the rabbit-*α*-spike antibody (Sino Biological, Beijing, China, 40592-T62; 1:1000 in 5% BSA/TBS-T) or human serum after vaccination (1:20 in 5% BSA/TBS-T) was used as primary antibody. Membranes were incubated with primary antibody for 1 h at room temperature, then washed five times for at least 5 min with TBS-T. Secondary antibodies used were either goat-*α*-rabbit-IRDye800CW antibody (LI-COR Biosciences, Lincoln, NE, USA, 926-32211; 1:15.000 in 5% BSA/TBS-T) or goat-*α*-human-IRDye680RD antibody (LI-COR Biosciences, Lincoln, NE, USA, 926-68078; 1:15.000 in 5% BSA/TBS-T). Membranes were incubated with the appropriate secondary antibody for 45 min at room temperature. After subsequent washing steps with TBS-T, membranes were documented with the LI-COR Odyssey^®^ CLx imaging system.

### 2.11. Isolation of Viral DNA for Quantitative PCR Analysis

Viral DNA from supernatant 48 h after infection of HEK293 cells was used for analyzing viral particle production using qPCR. Thus, the 96-well plate was subjected to three cycles of freezing and thawing to liberate viral particles from still-adherent cells. Supernatant after centrifugation (RT, 300× *g*, 5 min) was transferred to 1.5 mL reaction tube. Viral DNA was then isolated using a Quick-DNA Miniprep Plus Kit (Zymo Research Europe GmbH, Freiburg, Germany, D4069) following the instructions of the manual, and sample concentration was determined by UV spectroscopy. For qPCR, DNA samples were diluted to a concentration of 7 ng/µL and stored at 4 °C until measurement.

### 2.12. Isolation of Total RNA and cDNA Synthesis

Total RNA was isolated using TRIzol™ (Thermo Fisher Scientific, Waltham, MA, USA, 15596026) following the instructions of the provided manual. Cells seeded in 6 cm cell culture dishes were harvested in 1 mL TRIzol™. RNA samples were stored at −80 °C or immediately reverse-transcribed into cDNA using the PrimeScript™ first strand cDNA synthesis kit (Takara Bio Inc., Kusatsu, Japan, 6110A) following the manufacturer’s instruction manual. Prior to cDNA synthesis, 10 µg RNA was digested with 10 U DNAse I (Thermo Fisher Scientific, Waltham, MA, USA, EN0521) in MgCl_2_ buffer for 3 h at 37 °C. cDNA was diluted with 60 µL ddH_2_O for the analysis of mRNA splicing by PCR and stored at −20 °C until further use.

### 2.13. Quantitative PCR Analysis

Viral production was quantified by qPCR analysis. As such, 1.5 µL of isolated viral DNA was added to 10 µL TB Green Advantage Premix (Takara Bio Inc., Kusatsu, Japan, 639676), 0.2 µM of appropriate forward and reverse primer (E4 forward: 5′ TAGACGATCCCTACTGTACG 3′, E4 reverse: 5′ GGAAATATGACTACG TCCGG 3′, PLAT forward: 5′ AGGGCTGGAGAGAAAACCTC 3′, PLAT reverse: 5′ TTCCTTCACTGGCTCAGCTT 3′) and adjusted with ddH_2_O to a final volume of 20 µL. The following program was used for template amplification: cycle 1—95 °C, 1 min; cycles 2–46—95 °C, 10 s followed by 60 °C, 25 s. The E4 copy number of each sample determined by qPCR was normalized to PLAT (plasminogen activator, tissue type) concentration.

### 2.14. Bright-Field Microscopic Imaging

Prior to microscopic imaging, 3 × 10^4^ ACE2-overexpressing U2OS cells were transduced with spike-expressing HAdV-C5 vectors with an MOI of 200 for 72 h. When analyzing the effect of angiotensin II (AngII) on syncytia formation, ACE2-overexpressing U2OS cells (3 × 10^4^/well) were transduced with an MOI of 30–600 in the presence or absence of angiotensin II (2000 ng/well) for 72 h. Every 24 h, the medium was refreshed to consistently provide cells with AngII. Finally, 96-well plates were imaged using a Leica DMi8 S microscope.

### 2.15. Statistical Analysis

For statistical data analysis, the software RStudio was used. Normality was tested via the Shapiro–Wilk test and homogeneity of variance was investigated using Levene’s test. In the case of unequal variances, a Welch ANOVA was performed, and Dunnett’s T3 test was used as the post hoc test.

## 3. Results

### 3.1. Vector Design

**Figure 1:**
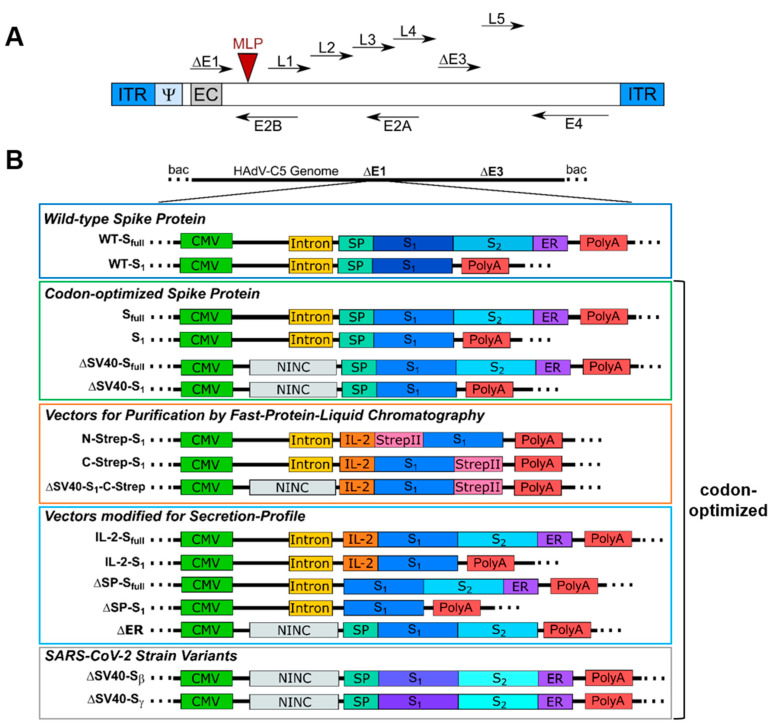
Design of expression cassette for all generated adenoviral vectors. (**A**) Schematic representation of human adenovirus type 5 genome used for this study. ITR: inverted terminal repeats, Ψ: packaging signal, EC: expression cassette, E1–E4: early genes 1–4, MLP: major late promoter, L1–L5: late genes 1–5. (**B**) All E1/E3-deleted type 5 adenoviral vectors were generated using homologous recombination in a bacmid context. WT: Wuhan-isolate spike protein, S_full_: full-length spike, S_1_: S_1_ domain, N-Strep: N-terminal StrepII-Tag, C-Strep: C-terminal StrepII-Tag, ΔSV40: replacement of SV40 intron with non-intronic non-coding sequence, NINC: non-intronic non-coding sequence, IL-2: IL-2 signaling sequence, SP: N-terminal spike protein signal peptide, ΔSP: deletion of N-terminal spike protein signal peptide, ER: endoplasmic reticulum retention signal, ΔER: deletion of ER retention signal, S_β_: *β*-variant of spike protein, S_γ_: *γ*-variant of spike protein.

For this study, an extensive panel of E1/E3-deleted type 5 adenoviral vectors with differences regarding their E1-located expression cassette was generated (Figure 1).

For a better overview, vectors were grouped based on functionality or properties. Most groups consist of vector variants expressing either the full-length spike protein (S_full_) or only the S_1_ domain, enabling differences in immune response. At first, the DNA sequence of the SARS-CoV-2 spike protein from the Wuhan isolate (NCBI reference sequence NC_045512.2) was inserted into an expression cassette consisting of a cytomegalovirus (CMV) promoter, simian virus (SV) 40 intron and polyadenylation signal (PolyA). In general, inserting introns upstream of a transgene coding sequence has been shown to enhance the expression of the transgene. The SV40 intron (see Table S3) has proven to enhance transgene expression level and transfection efficacy efficiently compared to other introns, such as the hCMV intron A [7]. As such, we decided to use this intron to enhance spike protein expression for our vaccine candidates. Subsequently, for the second group, the wild-type spike protein sequence was replaced with an human-expression system codon-optimized sequence harboring either the SV40 intron (S_full_ and S1) or a non-coding non-intronic sequence (ΔSV40-S_full_ and ΔSV40-S_1_). Moreover, a third vector group was designed to purify codon-optimized S_1_ protein using affinity chromatography. A StrepII tag was introduced, either N- or C-terminally (N-Strep-S_1_ and C-Strep-S_1_, ΔSV40-S_1_-C-Strep—codon-optimized). Furthermore, to study the effects of different domains of the spike protein on vector production and protein secretion, a fourth group of vectors was generated, where the N-terminal signal peptide of the spike protein was either deleted (ΔSP-S_full_ and ΔSP-S_1_) or exchanged with the interleukin (IL)-2 signal peptide (IL-2-S_full_ and IL-2-S_1_). One of the goals was to use the adenovirus vectors as expression platforms in cell culture to obtain significant amounts of correctly glycosylated spike protein. Previous studies showed that adenoviral vectors can be suitable for this purpose [8]. We used the IL-2 signal peptide, as in our experience with different proteins, it turned out that for efficient secretion of the target protein, the nature of the secretion signal peptide plays an important role, and in our hands, the IL-2 signal peptide has worked best so far (unpublished results). Additionally, the endoplasmic reticulum (ER) retention signal was deleted (ΔER). Lastly, a final group of viral vectors was generated in which the spike protein coding sequence was exchanged for codon-optimized sequences encoding the *β*- or *γ*-strain variants of the spike protein (ΔSV40-S*_β_* and ΔSV40-S*_γ_*).

### 3.2. Physical Characterization of Adenoviral Vectors

**Figure 2:**
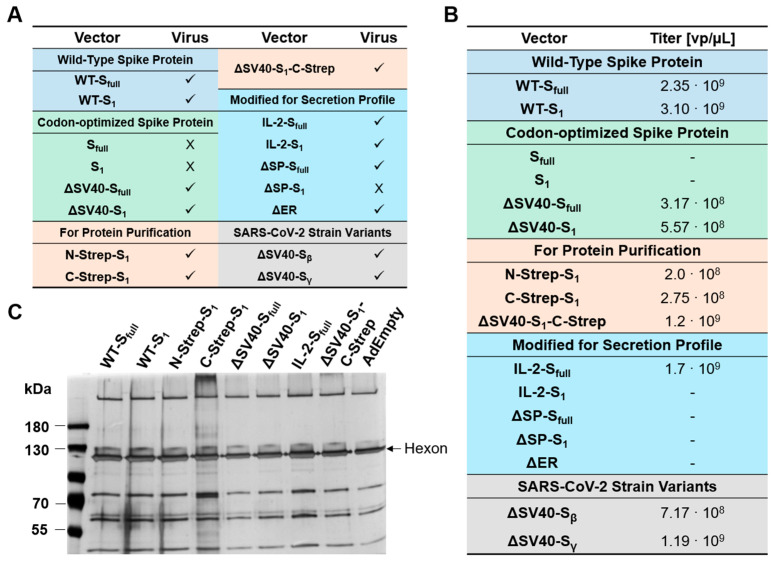
Characterization of adenoviral vectors. (**A**) Tabular listing of spike protein encoding vector constructs generating infectious viral particles. ✓: vector growth, X: vector growth not observed. (**B**) Physical titers of adenoviral preparations as determined after calculation using excitations at 260 nm by UV spectroscopic measurement. -: physical titers could not be measured. (**C**) Polyacrylamide gel was loaded with 5 × 10^9^ viral particles of preparations, proteins were stained using silver staining, and titers were adjusted by normalization to AdEmpty after determination of hexon band intensities.

To generate viral particles, E1-transcomplementing HEK293 cells were transfected with the linearized HAdV-C5 genome using PEI. While most vectors could be purified to moderate titers, some constructs were impaired in their ability to produce infectious viral particles (Figure 2). Noteworthily, viral particle production was either not possible (S_full_, S_1_, ΔSP-S_1_) or severely hampered (IL-2-S_1_, ΔSP-S_full_, ΔSP-S_1_) for most SV40 intron-containing constructs. Viral particle production was also hampered for constructs with deleted ER retention signal (ΔER). Due to low particle production, purification for these vectors was prematurely stopped after the first CsCl gradient and physical titers could not be determined (Figure 2B). For the remaining viral preparations, titers were adjusted by identifying hexon band intensity after separation of viral proteins by SDS-PAGE and subsequent silver staining (Figure 2C). With the exception of C-Strep-S_1_, no substantial differences in purity of viral preparations could be observed compared to AdEmpty (E1/E3-deleted vector with no transgene in expression cassette). As such, viral propagation seems to be affected by design of the ΔE1-located expression cassette.

### 3.3. Quantification of Viral Particle Production

**Figure 3:**
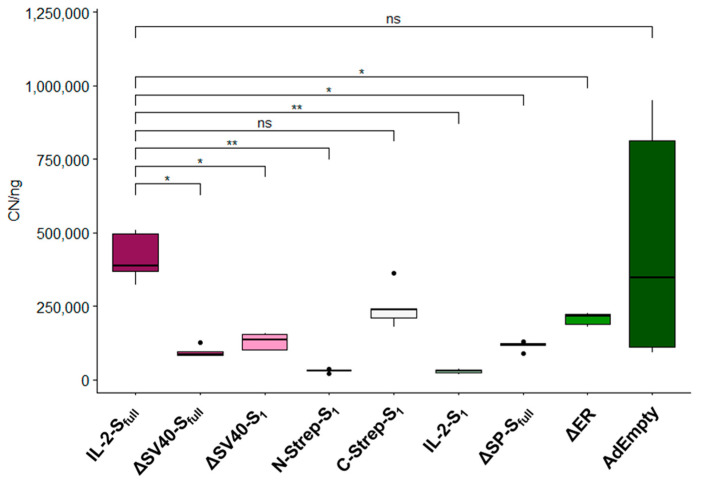
Characterization of infectious viral particle production. Quantification of infectious viral particle production of spike protein-encoding vectors by RT-qPCR after isolation of viral DNA from supernatant of HEK293 cells 48 h after infection. E4 copy number (CN) of each sample was normalized to PLAT gene in ng. Significance values were determined by Dunnett’s T3 test (*n* = 5; ns: *p* > 0.05, *: *p* < 0.05, **: *p* < 0.01).

Since several vectors seemed to be affected in their ability to produce infectious viral particles due to the design of their expression cassette (Figure 2), we further characterized infectious particle production by qPCR (Figure 3). As such, HEK293 cells were infected with an equal MOI of 200. Subsequently, viral DNA was isolated 48 h after infection and used for quantitative analysis. Infectivity was compared to IL-2-S_full_ because this vector showed growth behavior typical for Ad5 viral vectors (see Figure 2).

Compared to IL-2-S_full_ (416,800.2 ± 73,857.4), almost all vectors showed significant decreases in viral propagation based on detected isolated viral genomes (Figure 3). Although moderate titers were measured for both ΔSV40-S_full_ and ΔSV40-S_1_, the number of produced viral particles was decreased in comparison to the IL-2-S_full_ vector (ΔSV40-S_full_: 95,684.9 ± 16,736.2, *p* = 0.013, 4.35-fold decrease; ΔSV40-S_1_: 130,020.3 ± 25,406.0, *p* = 0.012, 3.21-fold decrease), indicating that viral propagation of these vectors was affected. N-Strep-S_1_ (30,299.7 ± 5574.5, *p* = 0.006, 13.76-fold decrease) as well as IL-2-S_1_ (28,864.1 ± 6901.5, *p* = 0.006, 14.44-fold decrease) appear to be especially hampered in their ability to produce infectious particles when compared to IL-2-S_full_. While not as pronounced as that with N-Strep-S_1_ and IL-2-S_1_, viral particle production for ΔSP-S_full_ (116,899.6 ± 13,954.9, *p* = 0.002, 3.57-fold decrease) and ΔER vectors (207,206.3 ± 19871.0, *p* = 0.002, 2.01-fold decrease) was reduced as well.

### 3.4. Protein Expression by Spike Protein-Producing Vectors

**Figure 4:**
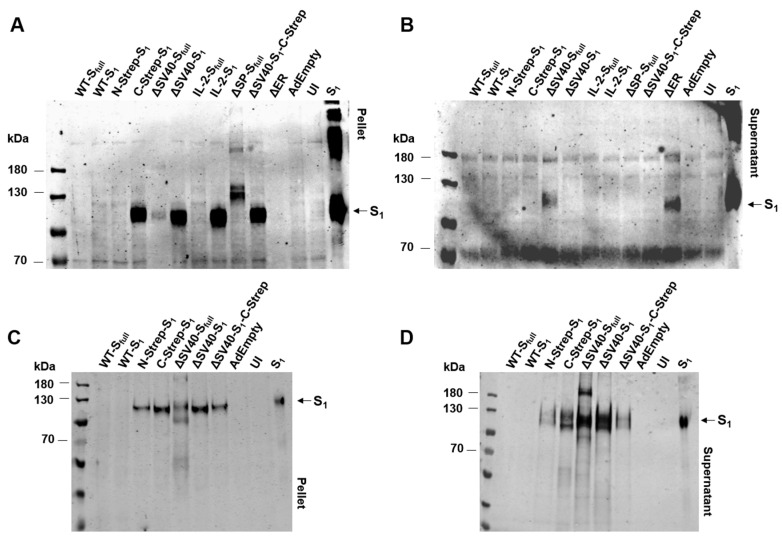
Spike protein expression after transduction with panel of spike protein encoding vectors. (**A**) Spike protein expression in cell lysates of U2OS cells after 48 h of transduction using rabbit polyclonal anti-S_1_ antibody. UI: uninfected cells. (**B**) Spike protein expression in supernatants of U2OS cells after 48 h of transduction using rabbit polyclonal anti-S_1_ antibody. Detection of spike protein expression in cell pellet (**C**) or in supernatants (**D**) of A549 cells after 48 h of transduction with a selection of vectors by incubation with human serum after boosting with mRNA vaccine.

For efficient use as a vaccine, transgene expression efficiency must be ensured and verified. Spike protein expression by the panel of HAdV-C5 vectors (wild type, codon-optimized vectors, vectors for protein purification and vectors modified for secretion profile) was determined by Western blot analysis (Figure 4). Purified S_1_ protein was also loaded onto the polyacrylamide gel as a positive control, whereas AdEmpty and uninfected cells served as a negative control. For a majority of constructs, such as C-Strep-S_1_, ΔSV40-S_1_, IL-2-S_1_, ΔSP-S_full_ and ΔSV40-S_1_-C-Strep, S_1_ expression at the expected molecular weight could be observed (S_full_ (glycosylated) ~180–200 kDa, S_1_ (glycosylated) ~120 kDa). Contrary to expectations, however, expression of the Wuhan-isolate spike protein (WT-S_full_ and WT-S_1_) could not be detected in either cell lysates or supernatants of transduced U2OS cells (Figure 4A,B) and A549 cells (Figure 4C,D).

Repeated experiments with different MOIs and different primary antibodies yielded no detection of wild-type spike protein expression. Additionally, protein expression could not be observed in cell lysates or supernatants of U2OS cells transduced with N-terminally StrepII-tagged codon-optimized S_1_ expressing vector (N-Strep-S_1_), as well as the IL-2-S_full_ vector.

While spike protein could not be detected in cell lysates of U2OS cells transduced with ΔSV40-S_full_ and ΔER vectors, protein expression could be discerned from supernatant. Syncytia formation was prominent in ACE2-overexpressing U2OS cells after transduction with ΔSV40-S_full_ and ΔER vectors, resulting in spike protein ending up in supernatant due to cell death. Furthermore, by incubation of membranes with human serum after boost vaccination, spike protein-specific bands could be detected in supernatants and cell lysates of transduced A549 cells for ΔSV40-S_full_ and ΔSV40-S_1_, as well as weakly for N-Strep-S_1_, C-Strep-S_1_ and ΔSV40-S_1_-C-Strep vectors (Figure 4C,D). Unexpectedly, the StrepII-tagged vectors showed lower protein expression compared to the ΔSV40-S**_1_** vector, although C-Strep-S_1_ and ΔSV40-S_1_-C-Strep vectors were successfully used for purification of codon-optimized S_1_ protein by fast protein liquid chromatography (see Section 3.6).

### 3.5. Analysis of mRNA Splicing of Vector Transcript

**Figure 5:**
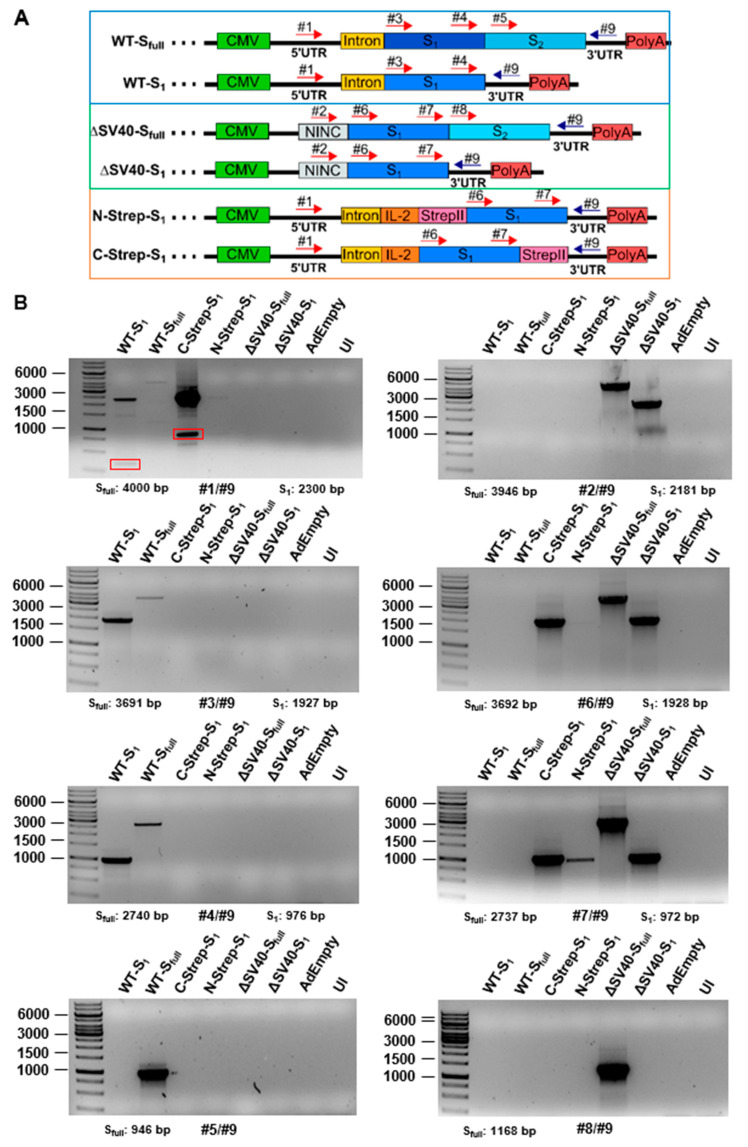
Analysis of mRNA splicing by PCR. (**A**) Schematic representation of primer placement for amplification of in cDNA-transcribed mRNA. (**B**) HEK293 cells were infected with a panel of viral vectors for 48 h and total RNA was isolated. RNA was reverse-transcribed into cDNA and then PCR was performed with appropriate primers (Table S2). PCR products were subsequently separated by agarose gel**.** Red boxes indicate bands excised for Sanger sequencing (see Figure S2).

Since we could not detect wild-type spike protein with our Western blot analysis and as we incorporated an RNA virus gene into a DNA background, we aimed to confirm the presence of expected mRNA transcripts expressed by different vectors using PCR. Analysis was performed by strategically positioning primers (Table S2) to amplify cDNA template after reverse transcription from isolated mRNA (Figure 5). As such, reactions were performed with forward primers being either located in the 5′ untranslated region (5′UTR) for SV40-containing vectors (#1), in the non-intronic non-coding sequence for ΔSV40-S_full_ and ΔSV40-S_1_ (#2) or in the S_1_ (#3, #4 for WT; #6, #7 for codon-optimized S protein) or S_2_ (#5 for WT; #8 for codon-optimized S protein) domain and a universal reverse primer located in the 3′UTR (#9).

Contrary to our expectations, the splicing of mRNA transcripts in A549 cells (Figure S1) appears to be unaffected, as PCR products in all primer combinations showed the correct length. Furthermore, no additional bands for any of the tested vectors were detected in all primer combinations. Unexpectedly, WT plasmid, which served as control, generated a PCR product with the forward primer located in the non-coding linker region while not being amplified with the 5′UTR primer (Figure S1). As for HEK293 cells, mRNA splicing seems to be affected mostly for SV40 intron-containing constructs (Figure 5). For primer combination #1/#9 (forward: 5′UTR, reverse: 3′UTR), multiple bands with lesser intensity were detected for WT-S_1_, WT-S_full_ and C-Strep-S_1_ vector. Also, contrary to expectations, no bands could be detected for N-Strep-S_1_ (Figure 5) in most primer combinations. Sequencing of these additional bands observed for C-Strep-S_1_ and WT-S_1_ (Figure S2) suggests the removal of SV40 intron in addition to IL-2 signal peptide and the majority of the S_1_ protein.

### 3.6. Purification of S_1_ Using Fast Protein Liquid Chromatography

**Figure 6:**
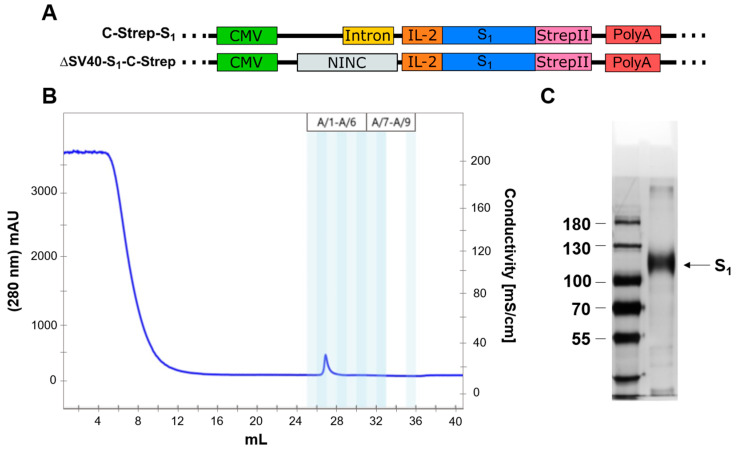
Purification of S_1_ from A549 cell culture media. (**A**) Vector constructs used for purification of S_1_ from A549. (**B**) Chromatogram of S_1_ purification from A549 cell culture media after 48 h of incubation using affinity chromatography (StrepII-StrepTactin™ system). (**C**) Polyacrylamide gel was loaded with 250 ng of purified S_1_ protein and stained utilizing silver staining. S_1_ protein band is annotated with an arrow.

For the expression of proteins that are posttranslationally modified in mammalian cells, a mammalian expression system is preferred. Purifying S_1_ from lung cells would ensure correct folding and glycosylation of the protein, which is why we aimed to accomplish the production of S_1_ in A549 cells. For this purpose, a panel of vectors was generated, in which codon-optimized S_1_ protein was fused to the StrepII-affinity tag (Figure 6A). Media of transduced cells with either C-Strep-S_1_ or ΔSV40-S_1_-C-Strep vector were collected. Prior interception of biotinylated proteins by the addition of avidin, cell debris and other impurities were removed by two centrifugation steps. Subsequently, StrepTactin™ columns (Cytiva, Marlborough, MA, USA, 29048653) were used for the purification of S_1_ protein via FPLC.

A total of 90 µg of S_1_ protein could be purified from media of 10 × 15 cm A549 cell culture dishes transduced with ΔSV40-S_1_-C-Strep (Figure 6B,C). Migration of the S_1_ protein differs from the calculated molecular weight of around 78.5 kDa, which suggests glycosylation of protein in A549 cells (Figure 6C). The functionality of purified S_1_ could be demonstrated on enzyme-linked immunosorbent assay (unpublished data).

### 3.7. Fusogenicity in ACE2-Overexpressing Cell Lines

**Figure 7:**
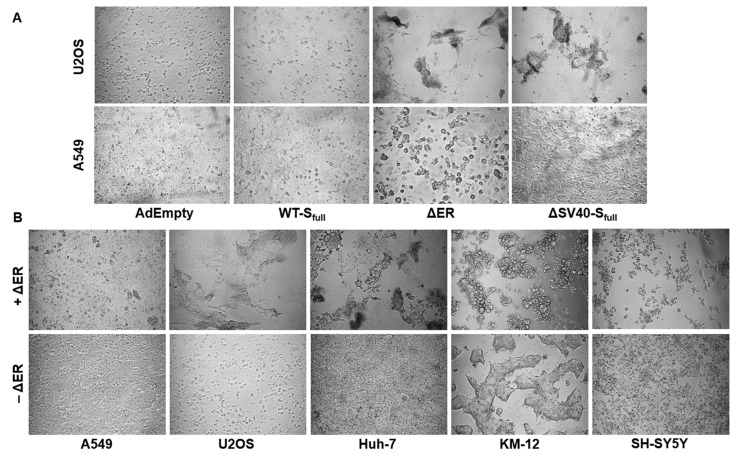

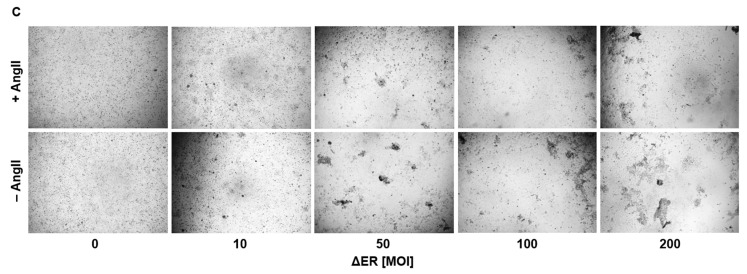
Microscopic Analysis of syncytia formation in ACE2-expressing cell lines. (**A**) ACE2-overexpressing U2OS cells were transduced with the aforementioned vectors with an MOI of 200. Syncytia formation was more pronounced when cells were transduced with the ∆ER vector. (**B**) The ∆ER vector is capable of inducing syncytia formation in a panel of different cell lines. (**C**) U2OS cells were pretreated with or without AngII, then transduced with ΔER with an MOI of 10–200. Syncytia formation is ACE2-dependent, as treatment with AngII decreases syncytia formation in ACE2-overexpressing U2OS cells. Magnification 50×.

In order to enter host cells, SARS-CoV-2 virus employs membrane fusion after attachment of spike protein to the foreign angiotensin-converting enzyme-2 (ACE2) receptor [9,10]. The spike protein plays a pivotal role in membrane fusion, acting as a fusogen and promoting membrane approximation by inserting its fusion protein after cleavage into S_1_ and S_2_ domains into the host cell membrane. To determine, whether spike protein expressed by vaccine vectors promotes cell fusion, ACE2-overexpressing U2OS cells were transduced with HAdV-C5 vectors for 72 h and subsequently imaged by bright-field microscopic imaging (Figure 7A). Compared to the control (AdEmpty) and Wuhan-isolate spike protein-expressing vector (WT-S_full_), the vector with deleted ER retention signal (∆ER) showed enhanced syncytia formation. While this effect could also be observed for SV40-S_full_ vector, it appeared less pronounced. This effect of enhanced syncytia formation by the ∆ER vector could also be witnessed for a panel of different cell lines, such as Huh-7, KM-12 and SH-SY5Y (Figure 7B).

ACE2 plays an essential role in the renin–angiotensin–aldosterone system (RAAS) by cleaving angiotensin II (AngII) into the heptapeptide angiotensin-(1–7) (Ang1–7). As such, it balances out AngII effects, which include vasoconstriction, blood volume regulation and inflammation [11]. To prove the involvement of ACE2 in the syncytia formation, ACE2-overexpressing U2OS cells were transduced with the ΔER in either the presence or absence of the natural ACE2 ligand AngII. Media were refreshed every 24 h to provide AngII continuously. Compared to the non-treated control, decreased syncytia formation of AngII-treated cells was observed (Figure 7C). This observation appears especially noticeable for U2OS cells transduced with higher MOIs (50–200) of the ΔER vector.

## 4. Discussion

With the conditional market approval for the emergency use of several adenoviral vector vaccines (e.g., Vaxzevria, Jcovden) during the COVID-19 pandemic, worldwide awareness of viral vector therapeutics rose. Adenoviral vectors proved themselves as a reliable platform for emergency outbreak diseases, being able to be quickly modified as well as manufactured at high rates while eliciting robust immune responses. While protein subunit vaccines and inactivated viral vaccines potentially require adjuvants for efficient stimulation of immune response, immunogenicity of adenoviral vectors achieves sufficient activation of the immune system on its own [2,12,13]. Furthermore, while mRNA vaccines do not face splicing difficulties, their low stability demands storage at low temperatures, thus proving problematic for vaccination in developing countries.

On the contrary, certain adenoviral vector vaccines do not require cold chain storage [2,12,13]. Overall, the adenoviral vector platform and the LNP-RNA platform do in fact complement each other rather than compete with each other. Still, knowledge regarding efficient adenoviral vaccine design is limited. Most of the market-approved vectors employ tetracycline-regulated protein expression for antigen production, masking possible transgene-related effects on patients, as these effects cannot be observed during propagation. Additionally, to date, there has been little experience pertaining to the incorporation of genes from an RNA virus into a DNA. As such, we strove to share the lessons we learned while generating our own spike protein-encoding vaccines based on human adenovirus type 5. In our study, we focused on investigating the effects of transgene codon optimization, expression cassette building blocks, and deletion or replacement of transgene internal functionalities on viral propagation and protein expression.

As our study shows, the design of such adenoviral vector vaccines for expressing a specific transgene is no trivial matter. A delicate balance between expression cassette functionality, viral propagation and protein expression level is necessary. We demonstrated the effect of expression cassette design on viral propagation, protein expression and mRNA splicing. As such, the use of SV40 intron in expression cassettes to enhance protein expression [7] instead hampered the production of infectious viral particles considerably. Furthermore, the deletion of certain domains of the spike protein can influence infectivity and viral particle production, as observed for the ΔER vector. While deletion or replacement of the N-terminally located signal peptide of the spike protein did not seemingly interfere directly with viral propagation in this study, a precise statement cannot be provided, as vectors with deleted or replaced signal peptide also contained the SV40 intron. Still, our data suggest that careful consideration should be given to the insertion of additional sequences, such as introns or affinity tags, as minor adjustments may result in less infectious viral particles. Through qPCR analysis, these observations concerning viral propagation could be confirmed. Nearly all evaluated vectors showed a significant decrease in detected viral genomes compared to the IL-2-S_full_ vector.

Although vector growth did not appear to be affected for ∆SV40-S_full_ or ∆SV40-S_1_ based on physical titers, viral particle production still seemed to be hampered according to qPCR results. As such, the expression of the spike protein may affect vector growth. As for protein expression analysis by Western blot, the spike protein was detected only for vectors expressing the codon-optimized version of the spike protein.Codon optimization of transgenes for vaccination approaches thus appears beneficial regarding protein expression level. Problems with mRNA splicing were expected to be the cause for undetectable spike protein expression because of SV40 intron inclusion in the ∆E1-located expression cassette. While additional bands could be identified when HEK293 cells were infected with vectors for one primer combination (#1/#9), no additional bands were observed when A549 cells were transduced. However, for most SV40 intron-containing vectors (such as WT-S_1_, WT-S_full_ as well as C-Strep-S_1_ vector), splice variants seem to exist. Sequencing of these additional bands (Figure S2) suggests not only excision of introns from the processed mRNA but also the deletion of the IL-2 signal peptide and a major part of the S_1_ protein. When predicting splice acceptor sites with the SpliceRover online tool [14], a splice site (1473–1484 bp) could be predicted at the incised location. Similar results were obtained for the band at around 500 bp for the WT-S_1_ vector construct (data not shown). As such, using expression-enhancing building blocks such as introns should be considered carefully, especially when transgenes contain splice acceptor sites. Transfer of RNA proteins into a DNA background or codon optimization may introduce additional splice sites, which should be considered for vaccine development. For codon optimization, it was previously determined that potential splice sites increased compared to the Wuhan isolate in ChAdOx-1 (Vaxzevria) and Ad26.COV2.S (Jcovden) in silico [15]. The use of other introns, such as the HAdV-C5 tripartite leader intron instead of the SV40 intron, may alleviate the aberrant splicing we observed.

Furthermore, protein expression could not be detected for the secretion-modified IL-2-S_full_ vector. This could explain the growth behavior (Figure 3) of this vector in comparison to the other vectors of this group (IL-2-S_1_, ∆SP-S_full_ and ∆SP-S_1_), which showed considerably hampered viral production (IL-2-S_1_, ∆SP-S_full_) or no viral particle production (∆SP-S_1_). In general, vectors expressing increased levels of spike proteins, i.e., most of the vectors modified for their secretion profile (IL-2-S_1_, ∆SP-S_full,_ ∆SP-S_1_), appeared to be hampered in viral particle production as measured with physical titers (Figure 2) and qPCR analysis (Figure 3). As such, high expression of the spike protein also seems to affect viral propagation. This emphasizes the importance of balancing transgene expression and efficient viral vector production. Alternatively, transgene expression can be adjusted by choice of promoter [16], a system to repress transgene expression during vector production [17] or microRNA downregulation [18,19].

Unexpectedly, spike protein expression could not be detected for N-Strep-S_1_ on Western blot analysis with anti-S_1_ antibody (Figure 4A,B). Only a very faint signal was detected when incubating with human serum after a prime boost with an mRNA vaccine. Contrary to this observation, a signal for C-Strep-S_1_ and ΔSV40-S_1_-C-Strep vectors could be determined by Western blot analysis (Figure 4). As such, placement of Strep-tag at either the N- or C-terminus may also influence transgene protein expression. Previous studies clarified that placement of affinity tags at the N- or C-terminus should be considered carefully with regard to protein functionality and localization [20]. As most signal peptides, including the signal peptide of the spike protein, are located at the N-terminus, placement of an affinity tag at C-terminus for spike protein expression to ensure proper localization seems appropriate.

Additionally, in this study, deletion of the ER retention signal seemed to promote syncytia formation in ACE2-overexpressing cell lines. A previous study also documented this increased formation of syncytia formation by ER retention signal-deleted vectors, when hACE2-mCherry cells were cocultured with S-∆19-EGFP-A549 (ER retention signal-deleted vector) [21]. Compared to AdEmpty and ∆SV40-S_full_, cell fusion between ACE2-expressing cells was increased when cells were transduced with the ∆ER vector.

This effect could also be observed in a panel of different cell lines, such as Huh-7, KM-12 and SH-SY5Y. Treatment with AngII compared to untreated control suggests involvement of ACE2 in the fusion process, as syncytia formation is decreased in AngII-treated cells. However, due to only performing a single experiment, a specific conclusion cannot be drawn, as further experiments would have to be performed. For example, ACE2 expression could be downregulated with miRNA and be compared with a nonsense miRNA control to observe whether ΔER vector-mediated syncytia formation would decrease.

To summarize, this study proved that the design of expression cassettes in adenoviral vaccine vectors can affect viral propagation and transgene protein expression. We observed that codon optimization of transgene sequence enhances protein expression, whereas deletion and/or replacement of protein domains can promote negative effects (e.g., fusogenicity) and negatively affect vector production. Expression-enhancing elements such as introns can prove harmful to viral infectivity. Splice sites may be generated by contextualizing an RNA gene into a DNA background or codon optimization. Therefore, for future adenoviral vaccine vector design, we emphasize the consideration of four main areas: (I) codon optimization, (II) careful consideration of deletion or replacement of transgene domains, (III) careful analyses of potential novel splice sites, and (IV) careful consideration of promoter choice and inclusion of, e.g., expression-enhancing elements such as introns. Nevertheless, one may still expect the unexpected.

## Data Availability

Not applicable.

## Supplementary Materials

The following supporting information can be downloaded at https://www.mdpi.com/article/10.3390/v15112155/s1, Table S1: Primers and oligonucleotides used for homologous recombination; Table S2: Primers used for mRNA splicing analysis; Table S3: Sequence of elements used in HAdV-C5 expression cassette; Figure S1: Analysis of mRNA splicing in A549 cells. Figure S2: Results of splicing analysis by Sanger sequencing.
