# Supplementary material for: Insights from the Construction of Adenovirus-Based Vaccine Candidates against SARS-CoV-2: Expecting the Unexpected
Source: Viruses. 2023 Oct 25;15(11):2155. doi: 10.3390/v15112155 (PMC10675337; doi:10.3390/v15112155)
Supplement: Supplementary file 1 [file viruses-15-02155-s001.zip › viruses-2665338-supplementary.pdf]

Supplementary Material

Table S1. Tabular Listing of all used Primers and Oligonucleotides for Homologous Recombination. Homology arms are underlined, melting temperature annotated as T_M.

Incorporation	Direction	Homolgy	Sequence [5′ → 3′]	T_M [°C]
codon-opt S_{full}/S_1	Forward	Bacmid	AAATCAAAGAACTGCTCCTCAGTGGATGTTGCCTTTACTTCTAGGCCTGTGCCAC<br>CATGTTCGTCTTCCTGGTC	80.5
codon-opt S_{full}	Reverse	Bacmid	GCGGTTGGAATAATAGCGAGAACAGAGAAATAGCGGCAAAAATAATACCCTTA<br>GGTGTAATGCAGCTTCACGCC	78.8
codon-opt S_1	Reverse	Bacmid	GCGGTTGGAATAATAGCGAGAACAGAGAAATAGCGGCAAAAATAATACCCTTA<br>CCGGGCTCTTCTGGGAGAGT	79.5
C-StrepII S_1 opt	Forward	Bacmid	GCAACTCCTGTCTTGCATTGCACTAAGTCTTGCACTTGTCACGAATTCGCAGTGCG<br>TCAATCTGACAACTCGG	80.6
C-StrepII S_1 opt	Reverse	Bacmid	GAAATAGCGGCAAAAATAATACCCTTACTTCTCGAACTGGGGGTGGCTCCACCG<br>GGCTCTTCTGGGAGAGT	81.5
rpsL-neo for ΔSV40	Forward	Bacmid	GTACGGTGGGAGGTCTATATAAGCAGAGCTGGTTTAGTGAACCGTCAGATCGGC<br>CTGGTGATGATGGCGGGATCG	83.0
rpsL-neo for ΔSV40	Reverse	Bacmid	GCACTGTGAGGAGACCAGAGGCAGCAGGACCAGGAAGACGAACATGGTGGCTC<br>AGAAGAACTCGTCAAGAAGGCG	84.1
ΔSV40	Reverse	Bacmid	GCACTGTGAGGAGACCAGAGGCAGCAGGACCAGGAAGACGAACATGGTGGCAC<br>CGGTGGATCCCGGGCCCG	87.3
rpsL-neo for ΔSP & IL-2- S_{full}/S_1	Forward	Bacmid	GAACTGCTCCTCAGTGGATGTTGCCTTTACTTCTAGGCCTGTGCCACCATGGCCTG<br>GTGATGATGGCGGGATCG	83.8
rpsL-neo for ΔSP &IL-2- S_{full}/S_1	Reverse	Bacmid	CTATTAGTATAAGCAGGTGGCAGCTGAGTCCGAGTTGTCAGATTGACGCACTGTC<br>AGAAGAACTCGTCAAGAAGGCG	81.1
IL-2-S_{full}/S_1	Oligo	Bacmid	GAACTGCTCCTCAGTGGATGTTGCCTTTACTTCTAGGCCTGTGCCACCATGTACAG<br>ATGCAACTCCTGTCTTGCATTGCACTAAGTCTTGCACTTGTCACGAATTCGCAGTG<br>CGTCAATCTGACAACTCGGACTCAGCTGCCACCTGCTTATACTAATAG	85.4
ΔSP-S_{full}/S_1	Oligo	Bacmid	GAACTGCTCCTCAGTGGATGTTGCCTTTACTTCTAGGCCTGTGCCACCATGCAGTG<br>CGTCAATCTGACAACTCGGACTCAGCTGCCACCTGCTTATACTAATAG	83.9
ΔSV40-S_β/ S_γ	Forward	Bacmid	CGAATTCTGCAGTCGACGGTACCGCGGGCCCGGGATCCACCGGTGCCACCATGT<br>TCGTCTTCCTGGTC	86.3
ΔSV40-S_β/ S_γ	Reverse	Bacmid	CGGTTGGAATAATAGCGAGAACAGAGAAATAGCGGCAAAAATAATACCCTTAG<br>GAGCCACAGCTACAGC	78.3

Table S2. Tabular Listing of Primers used for mRNA Splicing Analysis by PCR. Melting temperature annotated as T_M.

Primer	Orientation	Sequence	T_M [°C]
#**1** 5′UTR	Forward	GAGACGCCATCCACGCTGT	61
#**2** Linker fwd	Forward	GCAGTCGACGGTACCGCG	62.8
#**3** WT S_1 1 fwd	Forward	GGGACCAATGGTACTAAGAGG	59.8
#**4** WT S_1 2 fwd	Forward	GATCTCTGCTTTACTAATGTCTATGCA	60.4
#**5** WT S_2 fwd	Forward	GTTGAGGCTGAAGTGCAAATTG	58.4
#**6** opt S_1 1 fwd	Forward	CGGCACCAATGGCACAAAGC	61.4
#**7** opt S_1 2 fwd	Forward	CTGTGCTTTACCAACGTCTAC	57.9
#**8** opt S_2 fwd	Forward	CAGAATGTGCTGTACGAGAAC	57.9
#**9** 3′UTR rev	Reverse	GAGGCCGAGTTTGTCAGAAAGC	62.1

Table S3. Nucleotide Sequence for Elements used in HAdV-C5 Expression Cassette for Spike Protein Expression.

Element	Sequence
CMV Promotor	TCGTTACATAACTTACGGTAAATGGCCCGCCTGGCTGACCGCCCAACGACCCCCGCCCA<br>TTGACGTCAATAATGACGTATGTTCCCATAGTAACGCCAATAGGGACTTTCCATTGACG<br>TCAATGGGTGGAGTATTTACGGTAAACTGCCCACTTGGCAGTACATCAAGTGTATCATA<br>TGCCAAGTACGCCCCCTATTGACGTCAATGACGGTAAATGGCCCGCCTGGCATTATGCC<br>CAGTACATGACCTTATGGGACTTTCCTACTTGGCAGTACATCTACGTATTAGTCATCGCT<br>ATTACCATGGTGATGCGGTTTTGGCAGTACATCAATGGGCGTGGATAGCGGTTTGACTC<br>ACGGGGATTTCCAAGTCTCCACCCCATTGACGTCAATGGGAGTTTGTTTTGGCACCAAA<br>ATCAACGGGACTTTCCAAAATGTCGTAACAACTCCGCCCCATTGACGCAAATGGGCGG<br>TAGGCGTGTACGGTGGGAGGTCTATATAAGCAGAGCTCGTTTAGTGAACCGTCAGA
SV40 Intron	GTAAGTTTAGTCTTTTTGTCTTTTATTTCAGGTCCCGGATCCGGTGGTGGTGCAAATCAA<br>AGAACTGCTCCTCAGTGGATGTTGCCTTTACTTCTAG
IL-2 Signal Peptide	TACAGGATGCAACTCCTGTCTTGCATTGCACTAAGTCTTGCACTTGTCACGAATTCG
StrepII Tag	TGGAGCCACCCCCAGTTCGAGAAG

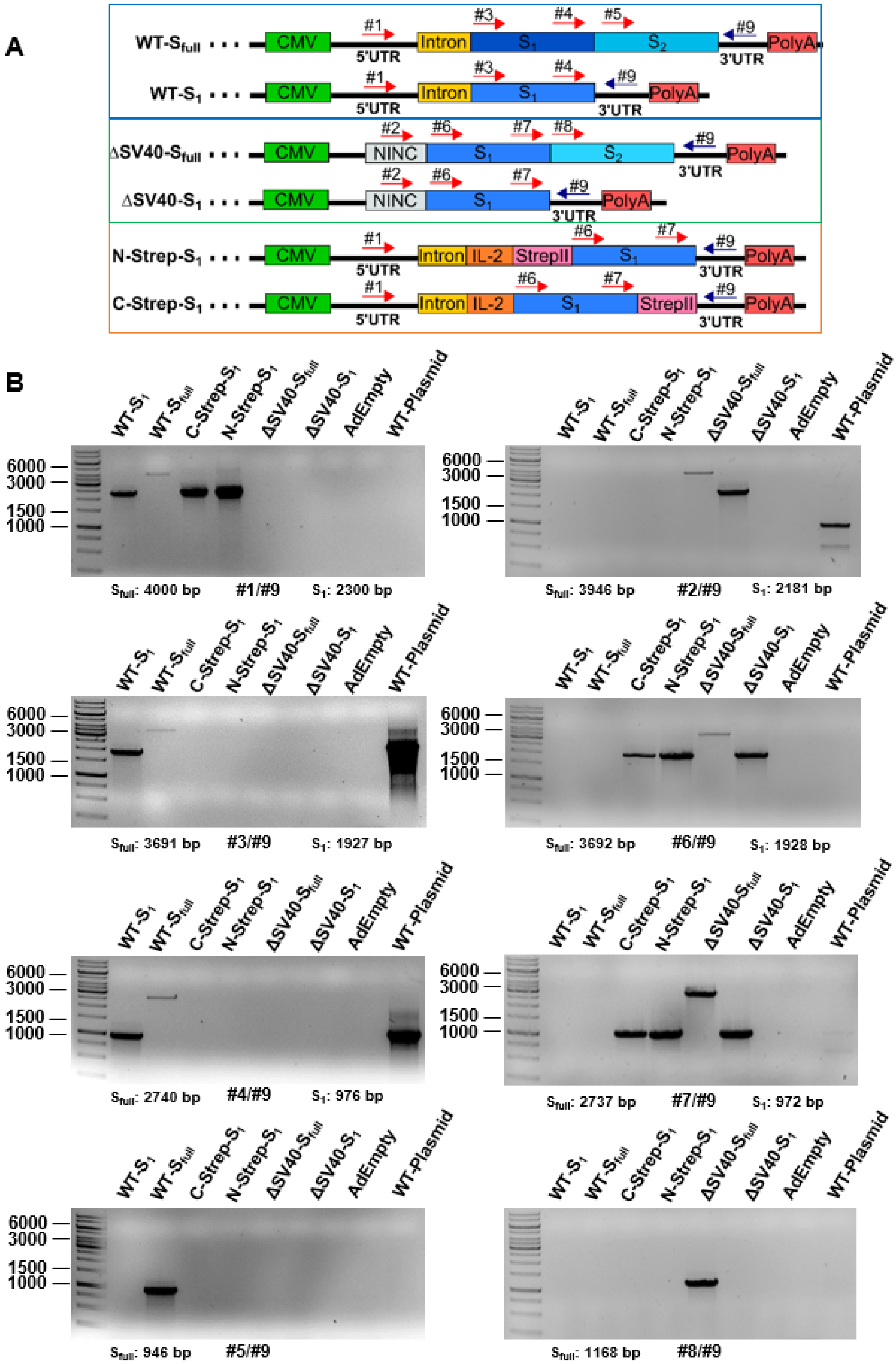

Figure S1. Analysis of mRNA Splicing by PCR. (**A**) Schematic representation of primer placement for amplification of in cDNA transcribed mRNA. (**B**) A549 cells were infected with a panel of viral vectors for 48 h and total RNA was isolated. RNA was reversely transcribed into cDNA, then PCR was performed with appropriate primers. PCR products were subsequently separated by agarose gel.

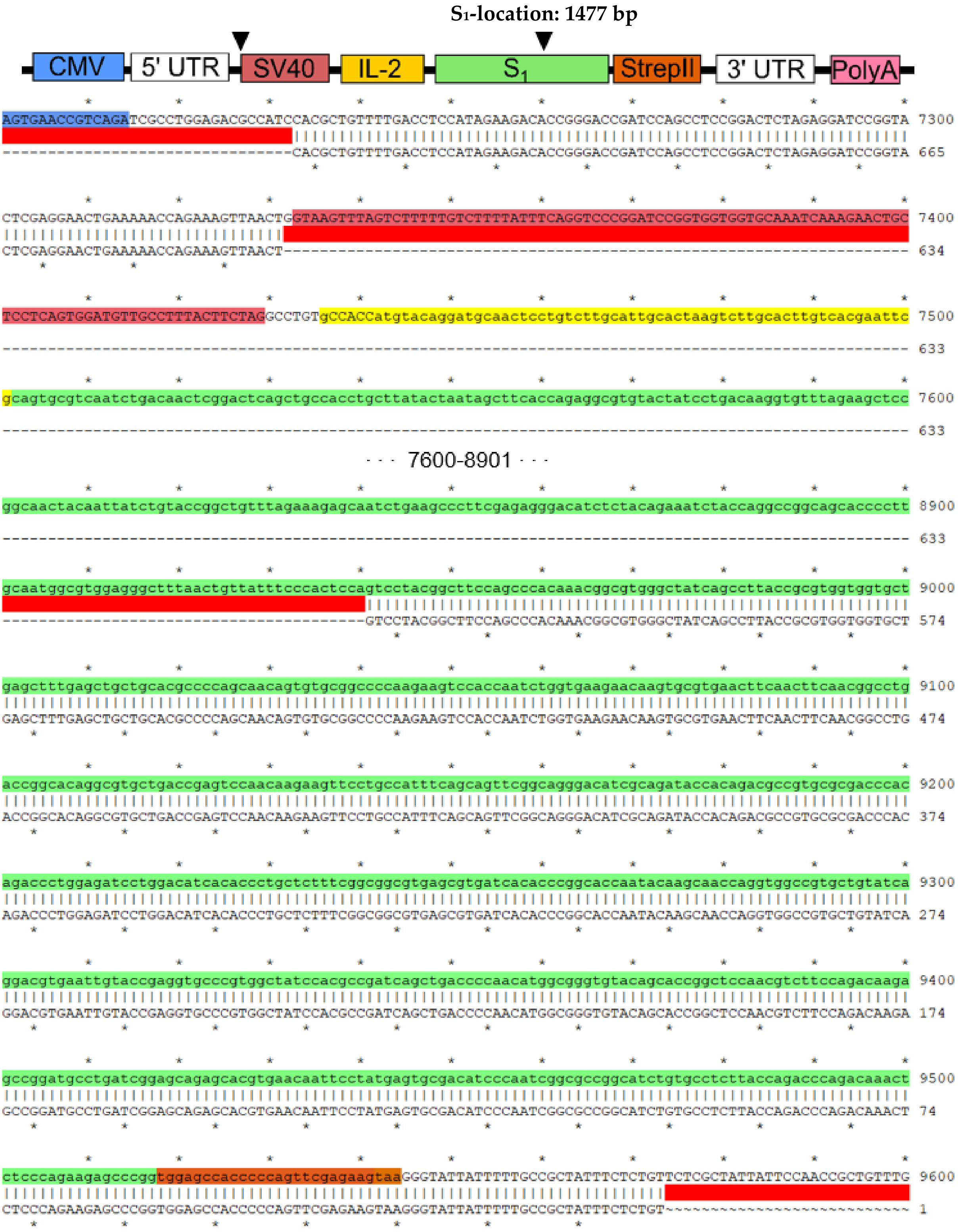

Location	Acceptor Site	Score
1473-1484	ACTCCAGTCCTA	0.89133

Figure S2. Results of Splicing Analysis. Additional band at around 1000 bp from C-Strep-S1 (Figure 5) was excised from agarose gel, DNA was extracted using phenol-chloroform extraction and subsequently analyzed via Sanger sequencing. Sequence alignment was performed using the plasmid editor ApE (https://jorgensen.biology.utah.edu/wayned/ape/). Splice Acceptor site prediction was performed using the SpliceRover Splice Site Predictor tool (http://bioit2.irc.ugent.be/rover/splicerover).
